# Self-reported needs for improving the supervision competence of PhD supervisors from the medical sciences in Denmark

**DOI:** 10.1186/s12909-017-1023-z

**Published:** 2017-10-23

**Authors:** Rie Raffing, Thor Bern Jensen, Hanne Tønnesen

**Affiliations:** 1 WHO Collaborating Centre, Clinical Health Promotion Centre, Bispebjerg and Frederiksberg Hospital, University of Copenhagen, Nordre Fasanvej 57, Hovedvejen, Entrance 5, 2nd floor, 2000 Frederiksberg, Denmark; 2 WHO Collaborating Centre, Clinical Health Promotion Centre, Faculty of Medicine, Department of Health Sciences, Lund University, Skåne University Hospital, Södra Förstadsgatan 101, 214 28 Malmö, Sweden; 3 Hospital of Southern Jutland, Department of Regional Health Research, University of Southern Denmark, J.B. Winsløws Vej 19, 3, 5000 Odense, Denmark

**Keywords:** PhD supervision, Education program, Clinical setting, Health science, Management, Medical context

## Abstract

**Background:**

Quality of supervision is a major predictor for successful PhD projects. A survey showed that almost all PhD students in the Health Sciences in Denmark indicated that good supervision was important for the completion of their PhD study. Interestingly, approximately half of the students who withdrew from their program had experienced insufficient supervision. This led the Research Education Committee at the University of Copenhagen to recommend that supervisors further develop their supervision competence. The aim of this study was to explore PhD supervisors’ self-reported needs and wishes regarding the content of a new program in supervision, with a special focus on the supervision of PhD students in medical fields.

**Methods:**

A semi-structured interview guide was developed, and 20 PhD supervisors from the Graduate School of Health and Medical Sciences at the Faculty of Health and Medical Sciences at the University of Copenhagen were interviewed. Empirical data were analysed using qualitative methods of analysis.

**Results:**

Overall, the results indicated a general interest in improved competence and development of a new supervision programme. Those who were not interested argued that, due to their extensive experience with supervision, they had no need to participate in such a programme. The analysis revealed seven overall themes to be included in the course. The clinical context offers PhD supervisors additional challenges that include the following sub-themes: patient recruitment, writing the first article, agreements and scheduled appointments and two main groups of students, in addition to the main themes.

**Conclusions:**

The PhD supervisors reported the clear need and desire for a competence enhancement programme targeting the supervision of PhD students at the Faculty of Health and Medical Sciences. Supervision in the clinical context appeared to require additional competence.

**Trial registration:**

The Scientific Ethical Committee for the Capital Region of Denmark. Number: H-3-2010-101, date: 2010.09.29.

**Electronic supplementary material:**

The online version of this article (10.1186/s12909-017-1023-z) contains supplementary material, which is available to authorized users.

## Background

Through this study, we have seen that supervisors of PhD Students in the Danish medical context report distinctive challenges caused by the circumstances related to performing research in a clinical setting and the fact that medical students in Denmark have little training in academic writing. Supervisors at the Graduate School of Health and Medical Sciences, Faculty of Health and Medical Sciences, University of Copenhagen, report that they struggle to cope with these challenges, amongst others.

Interestingly, up until recently, the role of supervisor has often been learned through experience [1–3], but today, universities offer more formal sources of learning. Consequently, PhD supervisors in Europe are moving from an experience-based apprenticeship to professionalization [4]. The consequences of insufficient supervision is poor completion rates as well as poor thesis quality [5].

Recent studies have shown that PhD supervisors desire a greater focus on the quality of PhD supervision [2–4, 6–8]. This is facilitated by the Bologna Process, which targets the development of tools to connect national education systems to facilitate the recognition of academic qualifications and exchanges among institutions [9]. One important principle was the role of supervision: “The crucial role of supervision and assessment: with respect to individual doctoral candidates, arrangements for supervision and assessment should be based on a transparent contractual framework of shared responsibilities between doctoral candidates, supervisors and the institution (and, where appropriate, including other partners)” [10].

In a Nordic context, analyses from Sweden (1999) and Denmark (2007) show that a large proportion of PhD students did not feel that their supervision was of a sufficiently high quality [11]. Thirty-seven percent of the Swedish students reported that insufficient supervision had prolonged their PhD project [11], and an analysis of drop-outs among Danish PhD students in the health sciences showed that 54% “agreed” or “agreed very much” that supervision was weak or insufficient [6]. The quality of supervision was recognized as a major predictor of whether the PhD project was completed in a successful and timely manner by 95% of students in the Danish study [6]. This finding is supported by both Lee and Delamont, who points out that a poor relationship between supervisor and student is associated with poor completion rates [5] and might also be damaging to the quality of the thesis [5].

As a result, the Research Education Committee of the University of Copenhagen wanted to increase the completion rate for PhD students through the quality development of PhD supervision. New PhD supervisors should enhance their competence as supervisors within the first year of supervision, and experienced supervisors should be offered PhD competence development training in supervision on a voluntary basis [12].

In Sweden, a Swedish Higher Education Ordinance was passed, stating that in 2007, PhD students had the right to have a trained supervisor [13], thus making training for supervisors compulsory. This training takes the form of obligatory courses for new PhD supervisors — for example “Research Supervision – in theory and practice” at Stockholm University [14]. At the University of Gothenburg, the course on Supervision in Postgraduate Programmes includes, for example, students’ experiences, rules and regulations, individual postgraduate programme, research-based literature, commitments, the supervisory role and ethics [15]. In Denmark, PhD supervision enhancement is progressing rapidly. Almost all universities offer compulsory or voluntary PhD supervision course activities in some form, and a national network for the development of PhD supervision has been established [16].

However, the effect of supervision courses can be called into question. Swedish researchers found that the doctoral programmes reported roughly similar problems before and after the training programmes were initiated. The problems are mainly associated with the relationship between the PhD student and the supervisor, thereby corresponding to international results [8]. The reason for the disappointing results may be multi-factorial; however, based on the literature, two reasons appear to be of primary relevance: the supervisors’ needs and disciplinary differences.

First, training programmes for PhD supervisors may not take the needs of the supervisors into consideration sufficiently. This is supported by Lee, who has argued that courses primarily focus on giving advice or assigning tasks, which the supervisors must undertake [2]. Manathunga et al. found that supervisor enhancement activities that were conceptualised as training tended to focus on sharing information or participating in basic skill development [17], and the authors call for programmes that go beyond such training. A step towards highlighting the supervisors’ perspective is to ask supervisors themselves about the needs they have experienced as supervisors. The self-reported wishes and needs of PhD supervisors in the health sciences, especially in the clinical field, are sparsely documented, as also noted by Turner in her work on new supervisors’ experiences of doctoral supervision: “Given that our data imply that existing training and support structures appear inadequate, this raises a number of questions about how to better equip and support new doctoral supervisors: what do new supervisors need to know and how do they learn this; how are new supervisors best supported; what could new supervisors do to help themselves; what can the university and departments do by way of preparation for the job ahead?” (https://www.srhe.ac.uk/conference2010/abstracts/0104.pdf). However, a main challenge appears to be the relationship between the supervisor and the PhD student [8], including skills in handling the psychological aspects of the supervision relationship [18] and not meeting the expectations of the students [19].

Second, PhD supervisor training programmes may not take disciplinary differences into consideration. Expectations of a training programme might therefore be considerably different due to the supervisors’ disciplinary backgrounds, and supervisors from different disciplines might have different needs. Research is required to fill these knowledge gaps. As Bengtsen also notes: “Research into disciplinary-specific doctoral supervision is still rather scarce” [5].

As this paper demonstrates, PhD supervisors have stated that the clinical setting offers some distinctive challenges, which differs from other settings due to patient treatment always being the first priority for supervisors in clinical departments. The word “clinical” in this paper simply refers to the unique setting defined by patient involvement in a broad sense.

Based on the above arguments, the aims of this study were to identify the self-reported needs and wishes of a group of PhD supervisors at the Graduate School of Health and Medical Sciences, Denmark in general and, more specifically, of a subgroup of PhD supervisors within the clinical field.

PhD at Graduate School of Health and Medical Science.

PhD programmes and studies differ from country to country differ, and the national and social context has a strong influence on how each programme is set up and managed. Even in Denmark, the requirement for PhD programmes varies between the different universities, but a short description of the PhD programme at the Graduate School of Health and Medical Science at University of Copenhagen as described in the Faculty’s regulations is presented here (http://healthsciences.ku.dk/phd/guidelines/).

To be enrolled in the PhD programme, the applicant must have qualifications equivalent to a Danish two-year master’s degree (120 ECTS). The PhD programme is normally a three-year full-time programme (but part-time programmes with a minimum of 50% involvement or integrated programmes can be allowed), and 30 ECTS and enrolment in other research environments (often abroad) is required. The PhD student is assigned a principal supervisor and a co-supervisor. The student is expected to direct the project management, and a high level of independence is emphasized in the regulation. The scope and level of supervision is not clearly defined, but a minimum of three assessments are required during the three-year course. The PhD thesis may either be written as a monograph or a synopsis with manuscripts/papers included, and the thesis is defended at an oral defence.

## Methods

### Design

An explorative approach has been applied to enable the PhD supervisors to address their needs and wishes for a supervision programme and to allow the investigator to ask more specific questions about these needs [20].

Since the aim of the study was to identify the themes that were most relevant to the interviewees, the data analysis was performed with an inductive thematic network approach whose primary purpose is “to allow research findings to emerge from the frequent, dominant, or significant themes inherent in raw data, without the restraints imposed by structured methodologies” [21].

### Setting

Twenty supervisors from a medical program at the Graduate School of Health and Medical Sciences at the Faculty of Health and Medical Sciences, University of Copenhagen, Denmark were interviewed.

### Participants

The supervisors chosen for interview constituted a purposive sample that aimed to represent variety in gender, age, experience, geography and disciplinary backgrounds (Table 1).

**Table 1:** Interviewees

Interviewee number	Gender	Age range	Disciplinary background	Employed in a clinical department	Experience as main supervisor of 2 students or less; 3 or more students
1	Woman	50-59	Medicine	Yes	3 or more
2	Man	50-59	Medicine	No	3 or more
3	Man	50-59	Medicine	No	3 or more
4	Woman	50-59	Medicine	No	3 or more
5	Man	30-39	Medicine	Yes	2 or less
6	Man	60-69	Odontology	No	3 or more
7	Woman	30-39	Human Biology	No	2 or less
8	Man	40-49	Medicine	Yes	2 or less
9	Woman	50-59	MA	No	2 or less
10	Man	40-49	Medicine	Yes	2 or less
11	Man	40-49	Bio Chemistry	No	2 or less
12	Man	30-39	Medicine	Yes	2 or less
13	Woman	60-69	Medicine	Yes	3 or more
14	Woman	–	Anthropology	No	2 or less
15	Woman	40-49	Odontology	No	3 or more
16	Man	–	Medicine	Yes	3 or more
17	Woman	40-49	Nursing	No	2 or less
18	Man	60-69	Medicine	Yes	2 or less
19	Woman	50-59	Nursing	No	3 or more
20	Woman	30-39	Medicine	Yes	2 or less

We included 20 PhD supervisors, including 10 women and 10 men from 37 to 66 years old. The supervisors had different disciplinary backgrounds: 12 medical doctors in various specialties, 2 dentists, 2 nurses, 1 human biologist, 1 master of arts, 1 biochemist and 1 anthropologist. Their positions included 2 institutional heads, 4 research directors, 6 professors, 2 senior scientists, 5 chief physicians, 2 lecturers, 1 department head, 1 staff specialist, 1 post-doctoral staff member and 1 resident (note that several of the interviewees held more than one position). Of the 20 supervisors, 9 were employed in a clinical department on a full or part-time basis. In terms of experience, 9 had been the main supervisor of 3 or more PhD students, and 11 had supervised 2 or fewer students.

### Procedure

The participants were identified through a list of PhD supervisors provided by the Graduate School of Health and Medical Sciences. The interviews lasted from half an hour to 2 h, with the main part of the interviews lasting about an hour. The interviews were conducted in the office or meeting room of the supervisors aside from two interviews, which were conducted at the WHO Collaboration Centre.

The development of the semi-structured interview guide included pre-interviews with administrative staff from the Graduate School, which is the administrative framework for the University of Copenhagen’s PhD program, as well as with PhD students. Prior to the interviews, the interview guide was tested in a pilot interview, which did not result in significant changes why the test interview was also included in the study. The interview guide included three research topics: factual information and the individual experience of being supervised; the supervisors’ experience of supervising others; and the supervisors’ needs and wishes for the programme in terms of improving competence. An additional text file provides more detail on this (see Additional file 1).

### Analysis

The interviews were recorded digitally and transcribed verbatim. The first part of the interview on factual information such as title, age, etc. was presented in a table in the transcription for a clearer overview. The analysis was inspired by the “Framework” model developed by Richie and Spencer, which allows both topics from the interview guide to be analysed along with additional responses. The *Framework* consists of five steps: familiarisation, identifying a thematic framework, indexing, charting and interpretation [22]. To strengthen the analysis, the construction of the thematic framework was undertaken as a group discussion by staff familiar with the data and involved in the project, such as interviewers, transcription staff and staff with teaching experience. The themes were selected according to their occurrence in the interviews. Based on the thematic framework, the transcripts were indexed and charted. Finally, the interpretation was guided by the research question and the themes emerging from the data, as recommended by Ritchie and Spencer [22].

## Results

### The need for a supervision programme

There was general interest in the programme, and several supervisors specifically asked to be contacted if we knew of a course being developed. Only five supervisors stated that they would probably not participate in a course. It was clear that some supervisors found it difficult to put their needs into words:

“*The problem is just that I do not know what I need. It is difficult for me to know. In a course for supervisors, it could be interesting to acquire a basic understanding of what they think a supervisor should know as well as how to develop the supervisors’ skills. Basically, I do not know what I need. One does not know*.” (Interviewee number 12).

### Requests

The supervisors expressed some clear needs and wishes regarding the programme. Through an analysis of the data material, seven overall themes with several levels of sub-themes were identified (Table 2).

**Table 2:** Results presented as themes and sub-themes, (*Expressed by PhD Supervisors from the clinical setting)

1) Formal requirements from the university
2) Responsibility of the supervisor a. Giving feedback b. Making sustainable agreements and appointments*
3) Problems that may arise a. Student-related i. Socially dysfunctional students ii. Inadequately qualified students (e.g., when writing a scientific article for the first time*; students requiring significant assistance) iii. Disruptions (Generally sickness and divorce and especially maternity/paternity leave) b. Process/study-related i. Miscommunication ii. Patient recruitment* iii. Conflicts, e.g., students vs. others (supervisors, co-workers, research partners) and between supervisors (personal interest, typically regarding publications)
4) Management a. Communication b. Psychological issues
5) Two main groups of students (Those aiming for research careers or other careers*)
6) Professional skills
7) Collegial group supervision

#### Formal requirements from the university

In addition to the formal requirements from the university, 12 of the supervisors requested instructions, definitions and guidelines related to their role as supervisors. The subjects mentioned were of an administrative nature and ranged from ministerial guidelines and employment conditions for students to the mandatory half year evaluations, students’ teaching obligations and courses for students. One supervisor wanted clarification of the rules because she had experienced how they were interpreted in different ways in practice:

*“…something about how the university system works, well the administrative requirements, because outside the university, so many things are interpreted in different ways.”* (Interviewee number 19)

In relation to this study, the Graduate School of Health and Medical Science has increased support for the supervisors in the last couple of years, and a booklet highlighting input for the role of the supervisor has been created (http://healthsciences.ku.dk/phd/hoejrebokse/ku-brochure/KU_god_vejledning_UK_2013_web.pdf). The main themes of the booklet are “Relationship between supervisor and PhD Student, Aligning Expectations, Clarity & Clear Communication, Ownership of the project, and the students’ function as a researcher and their personal educational journey”.

#### Responsibility of the supervisor

When asking the supervisors to define the difference between being the main supervisor and being the co-supervisor, many (10) mentioned responsibility as the key factor, with the main supervisor having more responsibility for the student and the project:

*“As main supervisor, one has more responsibility from the beginning for the content of the project, that it progresses as it should, and that the student obtains the data he or she needs. […] When you are a co-supervisor, you show up to the meetings and help with what you are being asked to help with.”* (Interviewee number 7)

Certainly not all, however, had a clear idea of the difference between being a main supervisor and a co-supervisor. Some thought that there was no difference at all; others spoke of co-supervisors having a more specific scientific or methodological perspective to contribute at certain points of the project. A few of the supervisors mentioned how, as a rule, the main supervisor had to be formally affiliated with the university. This resulted in some of the supervisors who were formally affiliated with the university to agree to become “pro forma main supervisors”.

Methods of giving feedback was a sub-theme of the responsibility of the supervisor. There were two tendencies: one was to tell the students in a concrete way what and how to revise their work. Another was to pose questions to the student to encourage him/her to reflect and thereby teach them how to arrive at the answers themselves:

*“I think it is a bit difficult verbally as well as in writing to say ‘well, this is not good enough; maybe you should go in this direction’ without saying ‘you must do this and that’. That is the easy solution […] but I do not think it is educational. I think it is better, that they reflect and conclude themselves.”* (Interviewee number 7)

Another sub-theme was the challenge of making plans and appointments with a busy clinical schedule. Supervisors argued that planned supervision could interfere with their clinical workday:

*“I think that in a clinical workday, with duties as well as unpredictable events unfolding, it is impossible to plan.”* (Interviewee number 8)

#### Problems

Another main theme that emerged was how to handle the different types of problems that could arise during a PhD study, either with the student or the research process. The supervisors gave several examples of problems they had experienced as PhD supervisors. The problems ranged from psycho-social issues, miscommunication, difficulties recruiting patients, conflicts involving students and/or (other) supervisors and disruptions to difficulties in deciding to what degree they should offer help to their students:

*“I have an example of a person where I was in doubt as to whether the person should receive the amount of help received, if it [the work] was good enough to defend and thereafter merited a PhD degree.”* (Interviewee number 10)

A sub-theme was that many students were inadequately qualified to write their first scientific article. Several supervisors mentioned how supervision during the PhD students’ first article was time-consuming. They were surprised at how poorly prepared they were, especially PhD students with a clinical background, for academic writing.

*“…there is a big difference in [the students’] point of departure when it comes to writing. There is especially a notable difference with medical doctors. Medical doctors are not trained in research when they come.”* (Interviewee number 4)

Another sub-theme specific to PhD projects in a clinical context was the challenge of recruiting a sufficient number of patients within a given time line.

*“The next step is patient inclusion. How do you decide when the inclusion is moving too slowly and when to say stop? The problem is when you decide that it is not working out after one and a half years, but then time is up…”* (Interviewee number 11)

#### Management

Management was another general issue. Some supervisors mentioned management tools such as communication or knowledge of how to work with psychologically challenging students or co-supervisors.

*“The main supervisor said that she saw some patterns in the student that were not normal, but I ignored this and thought that it was not fair to label somebody in that way. But then I reflected on what [the student] said, and I have researched those types of psychological patterns. How do you approach this issue as a supervisor?”* (Interviewee number 19)

#### Two main groups of students

The supervisors often divided students into two categories, depending on the students’ aim. The supervisors spoke of two basic groups of students having different aims with their PhD and therefore requiring different types of supervision. One group of students saw the PhD as a starting point for their research careers, while another group saw the PhD as a stepping stone for career advancement in another direction.

*“I think it is important to be engaged, to have a wish to do research and not just do it because you want a certain position in the future but because you think it is fun.”* (Interviewee number 1)*“Well, there are some PhD students where one can sense that it does not come from their heart — that it is something they feel they must complete to progress. […]. That they must have it on their CV to obtain a position, a final position or something.”* (Interviewee number 20)

It was obvious that the supervisors viewed those who worked toward a PhD to obtain a certain position as less motivated than those who aimed for a career in research.

#### Professional skills

Other supervisors requested knowledge of certain subject areas outside of their fields of expertise.

*“There are always some subject areas you know less about. There are different types of designs — for example, epidemiology and statistical lab experiments and experiments with animals. There are many facets. As a supervisor, you are often an expert in a single area”.* (Interviewee number 5)

#### Collegial group supervision (with experienced supervisors as moderators)

Finally, some supervisors (5) mentioned continuous collegial group supervision as an important element in a programme.

*“I think it would be a good idea to have a continuous forum for supervisors […] by which I mean an offer of a structured network where you could meet every two or three months […] I believe it would be a really good idea to have a facilitator in such a process.”* (Interviewee number 17)

### Special needs for PhD supervision within the clinical setting

As we have shown above, the clinical setting has additional requirements compared to a traditional supervising context. Two requirements relate directly to everyday life in the clinical department, where the needs of patients have the highest priority. This makes it difficult for supervisors to schedule time for supervision and comply with deadlines when patient inclusion is slow. Two requirements concern structural influences: the composition of the curriculum of the medical education, which does not include much training in writing, and the intense competition among medical doctors in Denmark for positions that cause them to pursue PhDs for career reasons.

## Discussion

The result of our study showed that PhD supervisors in the health sciences had unmet wishes and needs for improved competence regarding formal demands from the University and the responsibility of the supervisor in handling general problems and mentoring different groups of students, possessing leadership and professional skills as well as collegial group supervision. Four elements were specific to PhD supervisors in the clinical context: handling different groups of PhD students, problems with patient recruitment, writing the first article, and making sustainable agreements and scheduled appointments in a busy clinical setting. Among the wishes and needs identified in this study, the request for clarification of the formal demands of supervision and details regarding the supervisors’ responsibilities was surprising because guidelines and instructions are usually included in the descriptions of work roles. It would be both relevant and easy to develop and include these aspects in a PhD programme for supervisors in addition to the complementary subjects.

The most pronounced theme was handling general problems, which especially included the relationship between the students and the supervisor, amongst other sub-themes. The main challenge is that the PhD students and supervisors often have different expectations, needs and ways of thinking and working [23] as well as different learning and teaching styles [24], all of which could result in miscommunication and conflicts, as reported in the present study. In addition to the clarification of the rights and duties of the supervisors, inclusion of effective communication and related tools in a PhD supervisor programme may not only solve problems more effectively but also prevent their development.

Overall, PhD supervisors at the Faculty of Health and Medical Sciences, University of Copenhagen reported needs and wishes for improved supervision competence, including management and professional skills. The majority would participate in a PhD programme that included the themes and sub-themes identified in this study. Some supervisors even wanted to have continuous group supervision, e.g., every 3 months, that would be mentored by an experienced PhD supervisor.

### Challenges in the clinical setting

Interestingly, supervisors in the clinical field mainly those with a medical background — reported specific needs and wishes within this specific setting. They often have significant experience in combining research and clinical work with continuously shifting priorities that depend on more acute matters such as patients. Patients with acute needs are prioritized before supervision, and this has consequences for PhD students. Few supervisors have made plans and appointments with their students; instead, 10 of the supervisors mentioned that they had an “open door policy” and welcomed students at any time. This might cause problems, however, because shy and overly self-confident students would not seek supervision and may therefore risk becoming side-tracked. Based on the results of the present study, ad hoc supervision is not recommended and may require special training in an outreach supervision style.

Another element that turned out to be specific to the clinical context was supervision on how to write the first article. Although medical education and other health science programs often include written theses for bachelor and master programs or to obtain candidate status, better preparation for a research career with written publications is necessary. A combination of providing supervisors with the skills to supervise the first article and providing medical students with the necessary skills to write an article could be of relevance.

The third sub-theme relevant in the clinical context was patient recruitment. Difficulties with patient recruitment are specific to PhD supervision of patient-related projects, especially studies involving the inclusion of patients who are vulnerable to changes in health service structures, clinical procedures and poor learning curves, and communication and research skills of the project staff. Sufficient patient inclusion is crucial for timely completion of the clinical trials and therefore for the PhD study. Here, the restricted time frame of a PhD project collides with the relative unpredictability of patient inclusion.

Last, the two main groups of students was a relevant theme for a clinical supervision programme. A study from 2010 has shown a tendency among post graduate medical trainees to pursue PhD degrees to become more competitive for their future career and to increase their credibility within the field [25]. What concerned the supervisors was how to identify candidates who wanted to complete a PhD out of scientific interest and those who wanted the title to boost their CVs. Supervisors clearly preferred the scientifically interested candidates, whose interest was genuine and “came from the heart”, as one supervisor expressed it. We have not found this categorisation in the literature hitherto. It is important, however, to have the relevant competence to supervise both groups, and this theme should be included in the PhD programme.

The specific needs and wishes for the clinical PhD supervisors supported the approach that a PhD supervision programme should take into account the requirements of different professional specialities and backgrounds.

#### Strengths and limitations

The strengths of this study are the varied and large group of participants and the use of qualitative interviews that allow supervisors to express their wishes for a course in their own words. The limitations are of a socio- and cultural character. There might be other relevant needs in other cultural or disciplinary contexts where the relationship between the student and supervisor is of a different character. We did not distinguish between PhD students and those working toward a doctoral thesis, even though the two approaches to research may differ — for example, with regard to structural circumstances and motivations of the students. This might influence the results.

The next step would be to describe, establish and follow-up on a new PhD programme that integrates the results from this study. Further research should include interviews of PhD supervisors who have taken the programme as well as their PhD students to evaluate the effect of the new supervision programme. Other effect evaluations should include assessment of future drop-out rates and timely completion of PhD programmes.

## Conclusions

Most of the interviewed supervisors expressed the need for a course in supervision and requested that the curricula include the formal requirements of a supervisor, tools for how to manage problems arising in the supervision, management and, to some extent, the skills specific to their professional background. Several also mentioned that a course could include a session on how to facilitate collegial group supervision. The results of this study further add a clinical perspective to the work of developing a course for PhD supervisors. Here, the specific needs of the course curricula were how to make sustainable plans and appointments in a busy clinical environment, tools to supervise students with a medical background who are writing their first article, how to cope with slow patient recruitment within a fixed time frame, and how to manage groups of students with different levels of ambition and engagement.

We might assume that slow patient inclusion and making supervision appointments in a busy clinical environment in the medical context also applies to other geographic and cultural settings, whereas difficulties in writing the first article and the students’ motivation for a PhD depends on whether the local curricula includes writing skills and if the local competition is so intense that it requires a PhD to obtain a position in the desired geographical and disciplinary field.

We recommend that supervisors and PhD students, as well as the Graduate School of Health and Medical Sciences and relevant partners, participate in the continued development and improvement of supervision qualifications.

## Additional file

Additional file 1: Interview guide (PhD supervisors). The interview guide used when obtaining the data from the PhD supervisors. (PDF 66 kb)
