# Supplementary material for: Self-reported needs for improving the supervision competence of PhD supervisors from the medical sciences in Denmark
Source: BMC Med Educ. 2017 Oct 23;17:188. doi: 10.1186/s12909-017-1023-z (PMC5651628; doi:10.1186/s12909-017-1023-z)
Supplement: Additional file 1: — Interview guide (PhD supervisors). The interview guide used when obtaining the data from the PhD supervisors. (PDF 66 kb) [file 12909_2017_1023_MOESM1_ESM.pdf]

Interview guide (PhD supervisors)

Factual information

First I will ask you about some factual information

Name
Age
Disciplinary background
Position
How long have you been a PhD supervisor?
How many PhD students have you been the main supervisor of? Completed/ongoing
In addition, how many PhD students have you been the co-supervisor of? Completed/ongoing
Have you written a PhD thesis or a Doctoral thesis?
If a PhD thesis: How did you experience being supervised in the course of your own PhD

Experience with supervision

I will now ask you about your own experience with supervising

In your view, what is the difference between being a main supervisor and a co-supervisor?
Have you reflected on your own supervision style?
Have you reflected on how you give feedback?
What is good supervision to you?

We have talked to a group of PhD students about what was important to them in relation to supervision. An area of importance was, that supervisor and PhD students make explicit appointments (regular office hours, division of responsibilities, supervision style etc.) early in the course of the project.

Is that something you recognize?

How do you do the matching of each other's expectations?

Are there other issues, of which one should make explicit appointments?

Describe "The good PhD student" (to establish, what the supervisors find important, relation / subject knowledge)

What do you do in order to sustain the commitment of the PhD student in the course of the PhD project?

Is there a difference between how you perform supervision in the beginning and in the end of the course of a PhD project?

How do you prepare your supervision in the end of the process, when the student is about to become an expert in the area?

In order to prevent future PhD supervisors and PhD students from progressing the PhD project in a problematic direction, I would like to ask you, if you have experienced situations in the PhD supervision, where things have not worked out as you wished?

What went wrong?

Why did it go wrong?

How did it end?

How could the situation have been avoided?

Have you experienced, that the PhD administration has been involved in such a problematic situation?

- If no, why?
- If yes, how did it go?

Have you experienced, that there have been interruptions in the course of a PhD project due to leave of absence, illness, maternity / paternity leave etc. and how have you handled it?

The Course

I will now ask you some questions, relevant for the development of a future course

Are there parts of supervising, which have become easier with time and the number of PhD students?

Do you think a course could help overcome … (what they mention above)

Do you have any supervision competences, that you would like to enhance?

If you were to participate in such a course, what should it contain for you to find it relevant?

Could a part of the course be homework?

With regards to your experiences with less successful PhD procedures, are there elements which could be prevented in a course for PhD students?

What would you characterize as the criterion / the criteria of the successfully completed PhD procedure?
